# Evaluation of Bond Strength of Concrete Repaired Using Polyurethane Grout Material under Static and Impact Loads Coupled with Statistical Analysis

**DOI:** 10.3390/polym16192729

**Published:** 2024-09-26

**Authors:** Sadi Ibrahim Haruna, Yasser E. Ibrahim, Ali Al-shawafi

**Affiliations:** 1 Engineering Management Department, College of Engineering, Prince Sultan University, Riyadh 11586, Saudi Arabia; ymansour@psu.edu.sa; 2 School of Civil Engineering, Tianjin University, Tianjin 300350, China; ali91@tju.edu.cn

**Keywords:** polyurethane grout, concrete, bond strength, drop-weight impact test, repair

## Abstract

The effectiveness of repair work relies on whether the interface substrate can achieve sufficient bond strength when subjected to numerous stresses. This study investigated the bond properties of repaired normal concrete (NC-to-NC) elements, including cube, beam, and U-shaped specimens, after undergoing natural fracture due to flexural and tensile stresses. The specimens were repaired using a polyurethane (PU) matrix by gluing the two parts and applying compression, splitting, and drop-weight impact (DWI) tests to evaluate the bond strength properties. The results revealed that the PU matrix effectively repairs NC substrate with adequate bond strength, which exceeds the minimum allowable bond strength specified in the ASTM ACI 546-06 to rehabilitate damage concrete structures. The reference beams exhibit a peak applied load capacity of 15.6 kN with less deflection than the repaired samples. The compressive strength of the NC-to-NC repaired specimens loaded along and parallel to the interface plane revealed a decrease in compressive strength of 47.3% and 31.5% compared to the NC-R samples, respectively. The mean number of blows at the cracking stages appeared nearly equal for reference and repaired NC-to-NC specimens. The reference specimens exhibited an average number of 24 and 31 blows at the initial and failure stages, respectively, which were higher by 9.1% and 5.2% than the NC-to-NC repaired specimens. The PU binder showed promising results in achieving adequate interfacial bond strength under static and impact loads.

## 1. Introduction

Civil engineering infrastructure, such as concrete building, roads, and runways, are repaired and protected by preparing damaged areas and substituting them with cement-based materials [1,2]. The functions of the rehabilitated sections can deteriorate again due to cracks and the replacement of materials, leading to the perception of degradation of concrete elements [3]. Water can be a transporter agent for several aggressive substances [4]. Thus, appropriately limiting water from penetrating the repaired section and concrete is vital in achieving high durability. There are many polymeric resins, such as polyurethane resins, methyl methacrylate (MMA), epoxy resins, and urea-formaldehyde. Similarly, polymer modifiers repair concrete structures, including styrene-butadiene rubber (SBR) emulsion and epoxy resin (EP) [5,6]. PU is a complex polymer that exhibits good wear-resistance properties [7]. Cementitious materials modified with PU were reportedly used in a retrofitting project after seismic action due to their improved bending strength [8]. Polymer-based concrete is commonly utilized to repair structural elements where high durability and fast setting are needed [9]. Additionally, PU grout materials have been used in many repair works [10,11], road and runway facility repair [12], stabilization of expansive soil [13], and sandwich structures [14].

The interface bond strength of composites (NC substrate and repair material) is a significant factor in repairing damaged concrete elements. However, bond strength is remarkably influenced by many factors, such as NC compressive strength, moisture, curing technique, and surface treatment, as reported in past literature [12,13], and surface treatment affects bond behavior at the interface more than all other factors [15]. Li et al. [16] evaluate the bond properties between PU grout and NC. The authors investigated the influence of contact moisture on the interface. The result revealed a significant reduction in bond strength due to increased polyurethane density and moisture content at the interface. Most previous research has studied bond strength in static load conditions [17,18,19,20]. However, the repaired concrete is usually exposed to different impact loads under the serviceability state; for instance, bridge columns/piers and military structures are exposed to impact loads such as vehicular accidents and blast loads. For this reason, extensive investigation of the interface bonding behavior of repaired concrete structures involving normal concrete–ultra-high-performance concrete (NC-UHPC) and NC-NC under various load scenarios (static and impact) is crucial. Also, it aids in choosing a suitable material for rehabilitating and strengthening concrete structures.

Several testing methods have been used to determine the interface bond behavior between old and new concrete, including splitting tensile, flexural bending, slant shear, and pull-off tests [17,18]. Additionally, surface treatments provide sufficient bond strength between the NC substrate and repair materials. Previous studies have reported the application of many bonding agents for repairing concrete structures: these include silicate-based curing agents [21], expoxy resin [20], metakaolin based geopolymer mortar [22], and polymer-modified tack coat [23]. The concentrations of silicate-based curing agents on repaired concrete affect the interface bond strength between old and new concrete, as reported by [21]. Vishavkarma and Harish [24] developed foam-concrete-containing ground-granulated blast furnace slag (GGBS) and fly ash (FA) for the potential repair of concrete. The authors reported that the developed foam concrete revealed promising results, serving as an alternative concrete repair material, with comparable or superior tensile and bonding strength. Rousakis et al. [25] used deformable polyurethane joints and fiber grids for seismic actions in the evaluation of reinforced concrete. Yang et al. [19] investigated the flexural behavior of precast UHPC-NSC samples. Al-shawafi et al. [26] reported that interface surface treatment significantly improved the bonding strength in the composites NC-UHPC and normal concrete–polyurethane-based polymer concrete (NC-PUPC) specimens. Moreover, the NC substrate combined with PUPC repair materials demonstrated better bond strength than the NC-UHPC specimen under static and impact load conditions. Zhang et al. [27] considered the effect of interface morphology on the bond properties at multi-scale NC-UHPC subjected to flexural loads. The horizontal shear resulted in failure at the NC-UHPC interface. Wu et al. [28] analyzed the shear failure of the UHPC-NC interface using U-shaped studs. EL-Afandi et al. [29] conducted a compressive review on NC-to-NC bond strength. They reported that shrinkage properties, concrete type, surface treatment, and concrete compressive strength affected the bond strength. The efficiency of repair work relies on the causes of damage and the chosen repair method. Effective long-term repair techniques address the primary causes of cracks. Therefore, the aim of this research was to assess the capability of polyurethane grout in repairing concrete elements, including cube, beam, and U-shaped specimens. The bond strength of the repaired NC-to-NC composites was evaluated using static and dynamic loads. Experimental programs, including compressive, flexural, and multiple drop-weight impact tests, were developed. Moreover, the statistical approach was used to evaluate the variability of the impact strength data.

## 2. Materials and Methods

### 2.1. Materials

**Figure 1:**
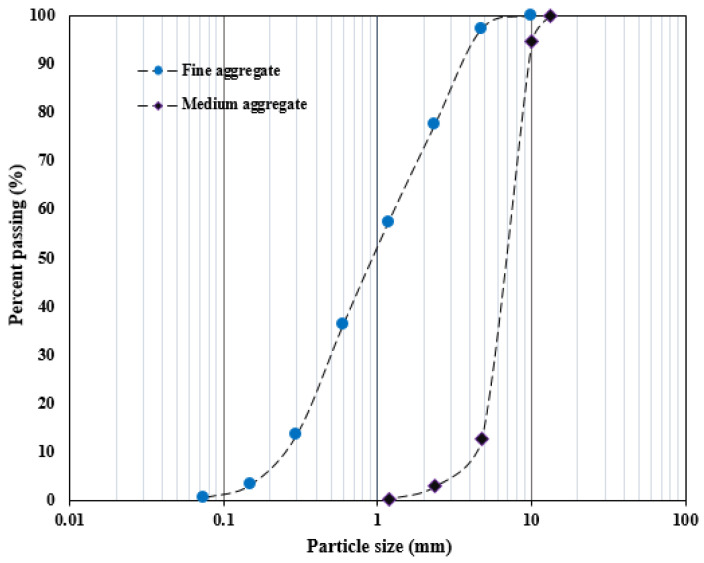
The gradation curve of aggregate used in this study.

The concrete mixtures were produced by mixing grade 43.5 R ordinary Portland cement (OPC), medium aggregate, and sand. The cement used to prepare concrete complied with the requirements specified in Chinese national standard GB 175-2007 [30]. Natural river sand and crushed medium stone were used as fine and coarse aggregates with large sieved sizes of 4.75 and 10 mm, respectively. The mix proportion for preduction of the concrete mixture is summarized in Table 1. The sand had a fineness modulus 2.82 and an apparent density of 2626 kg/m^3^. The medium aggregate exhibited a 2600 kg/m^3^ density and a fineness modulus of 2.67, respectively. The aggregate particle gradation curve is depicted in Figure 1. A polycarboxylate-based superplasticizer revealing a 20% water reducer was incorporated into the mixture for workability.

**Figure 2:**
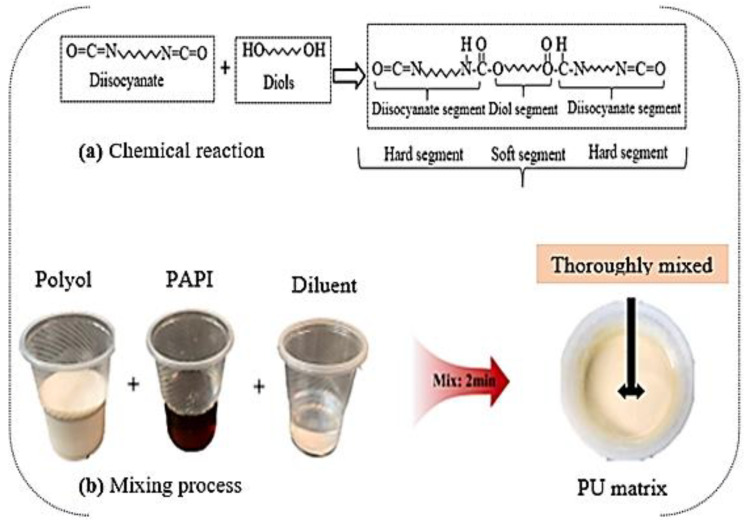
Synthetic procedure for the production of PU matrix.

The polyurethane (PU) mixture was formulated by combining diisocyanate involving the hydroxyl group (polyol) with polymethylene polyphenylene isocyanate (PAPI) in a 6:1 mix ratio by weight and thoroughly stirred in a cup. The PU diluent was introduced into the mixture to improve the PU matrix’s performance. The PU binder’s mix design is summarized in Table 2, and the chemical reaction and systematic procedure for producing the PU matrix are depicted in Figure 2a and b, respectively.

#### Sample Preparation

**Figure 3:**
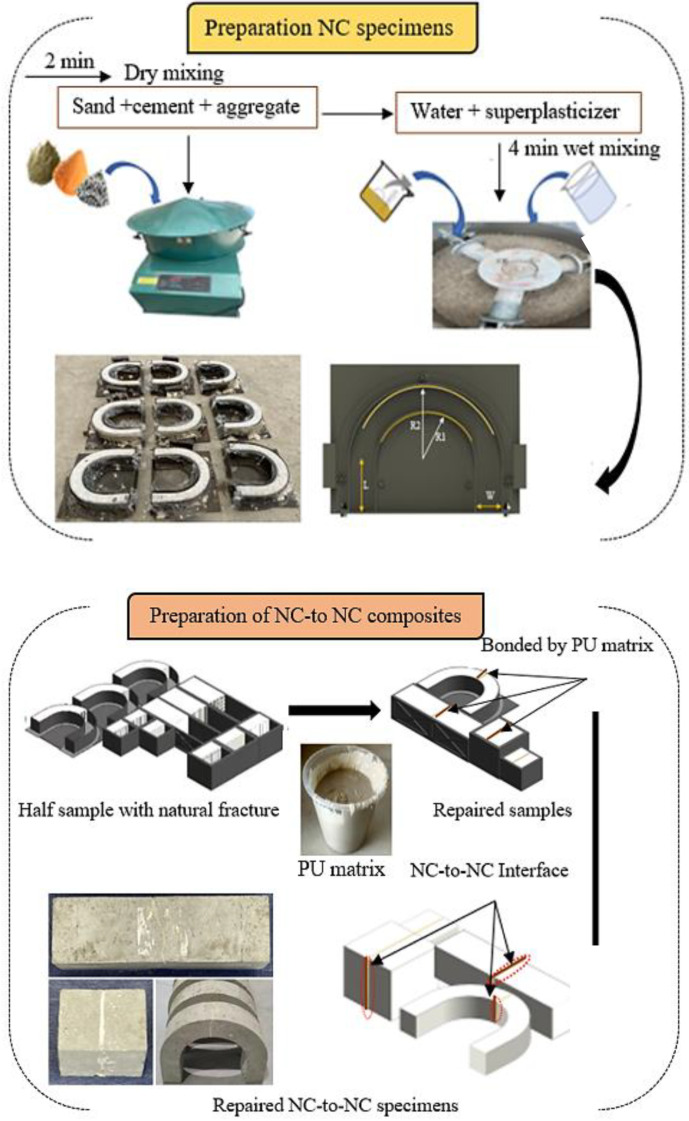
Schematic process of formulating the repaired specimens. Noted: U-shaped dimension: L = 85 mm, W = 50 mm, R1 = 85 mm, and R2 = 135 mm.

The NC mixture was produced following standard code JTG55-2011 [31]. The concrete mixture preparation involved combining sand, coarse aggregate, and cement in a concrete mixer, allowing them to mix to dry for about 2 min. A solution of water and superplasticizer was mixed thoroughly for 3 min to obtain a homogeneous mixture. The preparation method is shown in Figure 3. Subsequently, the prepared concrete was cast into various molds, including cubes, beams, and U-shaped specimens to determine the interfacial bond properties. A 150 mm cube, beam (100 × 100 × 400 mm^3^), and U-shaped specimen were used, cured for 28 d, and broken into two parts through natural fracture. Tensile and flexural stress were applied at the mid-sections of the cube, beam and U-shaped specimens, respectively. A PU binder was used to glue the interface surface of the two broken parts; the pictorial representation of the methods is depicted in Figure 3.

### 2.2. Testing Methods

#### 2.2.1. Impact Strength Test

**Figure 4:**
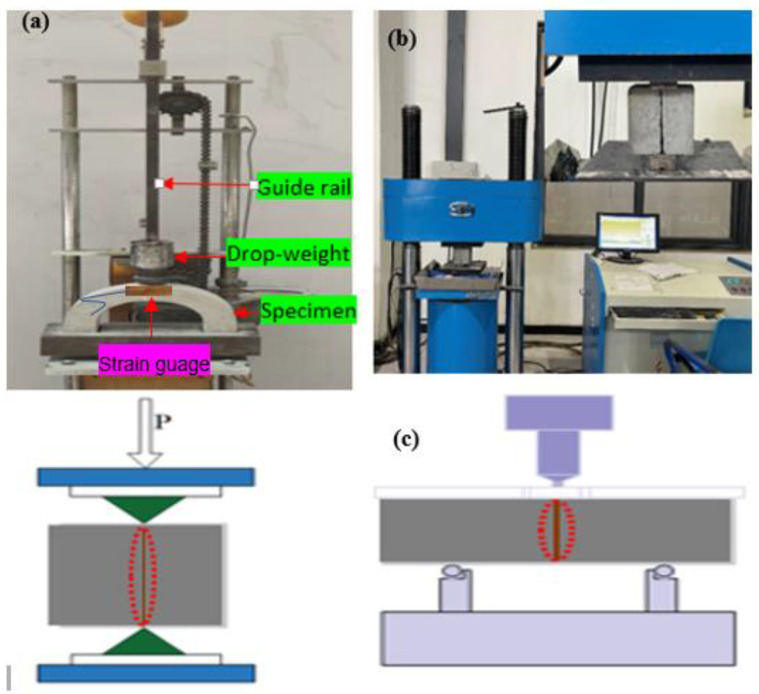
Experimental setup: (**a**) drop-weight impact test, (**b**) splitting tensile, and (**c**) compression and flexural tests.

The reference and repaired U-shaped specimens were exposed to impact loads through a DWI test, similar to the method found in [32], using a drop-weight of 0.785 kg, as shown in Figure 4a. The hammer weight repeatedly fell at 2 s intervals to stimulate the impact test. The test involved continuously dropping a mass from a constant height onto a test sample and recording the number of drops that cause an initial crack and ultimate failure. Impact strength data were measured at the initial cracking (N1) and failure strength (N2). The U-shaped specimens were equipped with a strain gauge with model number BX120-20AA-P15, featured with 120.0 ± 0.3Ω resistance. The strain gauge was pasted at the mid-section of the U-shaped specimen, corresponding to the interface location, to easily detect the first crack during impact testing.

#### 2.2.2. Compression and Bond Strength Test

A compression test of the NC-R and NC-to-NC specimens was conducted following 50081-2002 [33] using 100 cube samples. The average of three samples was considered as the concrete compressive strength. A UTM with a 20-ton loading capacity set a loading rate of 0.5 MPa/s. The interface bond strength properties of the repaired cube and beam specimens were studied through splitting, compression, and bending load, which were loaded at the interface plane, following ASTM C496 [34] for splitting and ASTM C78 [35] for the flexural test (see Figure 4b,c). The splitting tensile bond (*ft*) and flexural strength *Q* were calculated using Equations (1) and (2), respectively. The load–strain data of the test samples were concurrently measured by load–cell and strain gauge.

(1) $$ft=\frac{2P}{\pi A_{sp}}$$

(2) $$Q=\frac{3PL}{2bd^{2}}$$

*P*, *L*, *b*, and *d* are the failure load, clear loading span, width, and depth of the beam specimen, and *A_sp_* is the area of the bonding plane, in mm^2^.

## 3. Results and Discussion

### 3.1. Flexural and Compression Properties of the Composite Specimen

**Figure 5:**
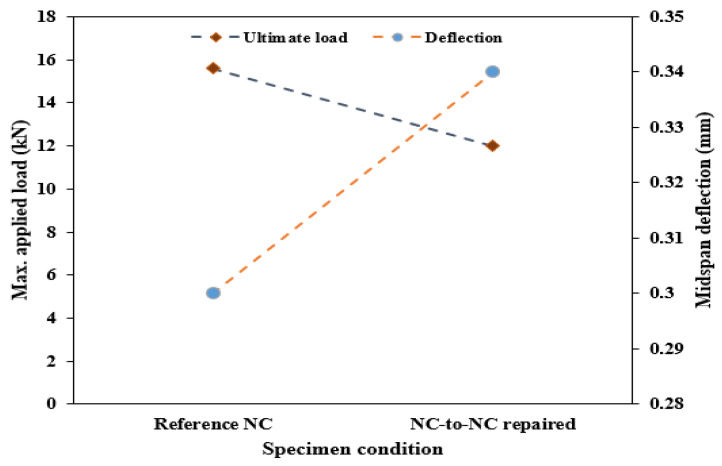
Flexural behavior of the beam specimen.

Figure 5 shows the flexural properties of the repaired beam specimens; the reference specimens exhibit the highest average load-carrying capacity with a peak load of 15.6 kN, with an induced deflection of 0.3 mm. The bonding ability of the PU binder used to join the fracture specimens shows little reduction in the flexural load capacity, with 12.0 kN representing a 24% reduction. However, these specimens revealed a higher average deflection of 0.34 mm than the reference specimen. This behavior is attributed to the elastic behavior of the PU binder. The average compressive strength of the three (3) NC-R specimens was found to be 42.5 MPa, while the compressive strength of the NC-to-NC repaired specimens loaded along the interface plane was 22.5 MPa, representing a reduction of 47.3% compared to the reference specimens. However, when the composite cubes were tested parallel to the interface plane, the compressive strength increased to 29.1 MPa, indicating a reduction of 31.5% compared to the reference specimen.

### 3.2. Bond Properties of the NC-to-NC Repaired Specimen

Table 3 compares the observed bond strength and acceptable lowest direct shear bond values at the interface of the composites (NC substrate and repair materials) based on ACI 546-06 [36], which highlights guidelines for studying the bond properties for an appropriate repair material in rehabilitating the impaired concrete structure. Table 3 shows that three cube samples were tested, and their average was regarded as the bond strength of the NC-to-NC repaired samples, which was found to be 2.8 MPa. The result indicated that the obtained bond strength between NC and NC substrate repaired with PU matrix is greater than the minimum acceptable value of bond strength for repair application at 28 days curing time, specified in ACI 546-06 [36]. Thus, it can be deduced that sufficient bond strength was achieved to repair concrete-to-concrete substrates using a PU matrix.

### 3.3. Impact Resistance of the Repaired U-Shaped Specimen

**Figure 6:**
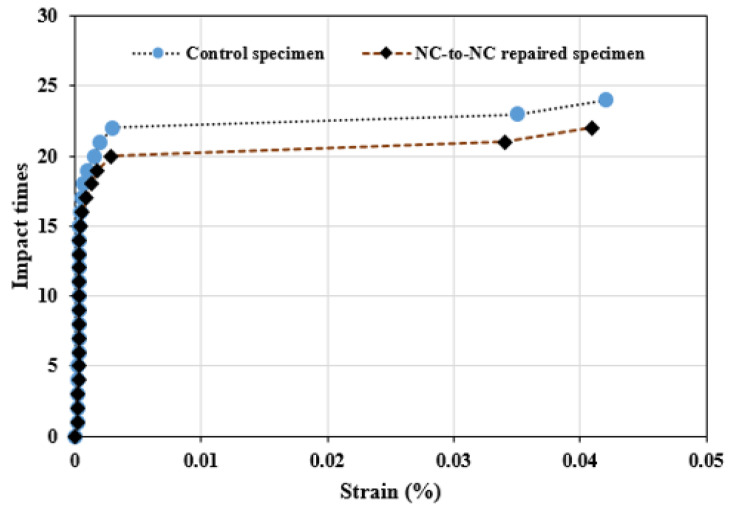
Impact blows vs. induced strain.

Figure 6 compares the impact test results of 20 specimens tested from the reference and repaired U-shaped specimens. The reference showed large strength deformation compared to the NC-to-NC repaired specimens. The reference specimens exhibit an average number of 24 blows and 31 blows at the first and failure stages, respectively, which are higher than that of NC-to-NC repaired specimens by 9.1% and 5.2%. The standard deviation of impact data ranges from 6.66 to 6.8, with a coefficient of variation of 22.23 to 30.3% for both cracking stages. The variation in the data between the reference and repaired U-shaped specimen is also demonstrated in Figure 6, which reflects the impact time against the induced strain due to the impacted multiple drop-weight. The results presented in Figure 6 represent those specimens selected from the two groups. The strain gauges were pasted on the specimens to detect the induced strain. A slight variation was obtained among the two specimens tested under this testing condition.

**Figure 7:**
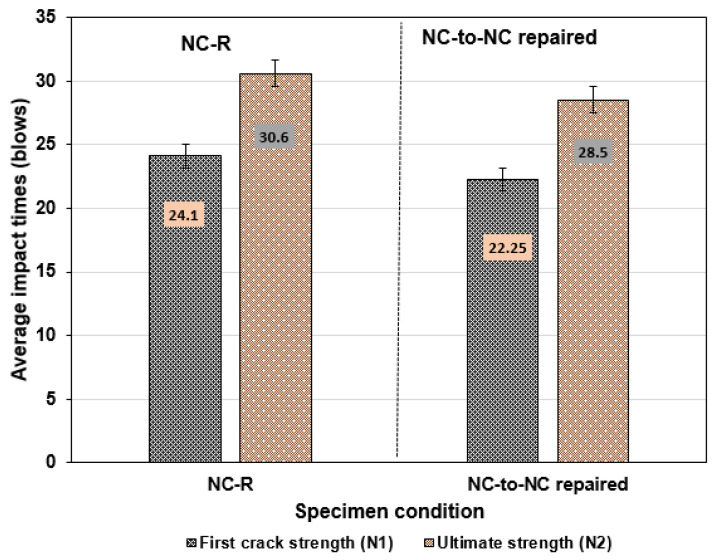
Comparison between NC-R and NC-to-NC repaired specimen under repeated loads.

According to the testing condition, the average impact times at the two cracking stages (N1 and N2) are presented in Figure 7. As can be seen, NC-R specimens exhibit the highest average impact times at both cracking phases, with N1 = 24 blows and N2 = 31 blows. The group of NC-to-NC repaired specimens showed a slight decrease in the average impact times with N1 = 22 blows and N2 = 29 blows. This result clearly illustrates the bonding ability of the PU matrix, which can resist repeated impact loads after repair application; hence, the bond strength at the interface of the composite is a vital issue in rehabilitating the impaired concrete elements.

#### 3.3.1. Impact Strength of NC-R Specimen

**Figure 8:**
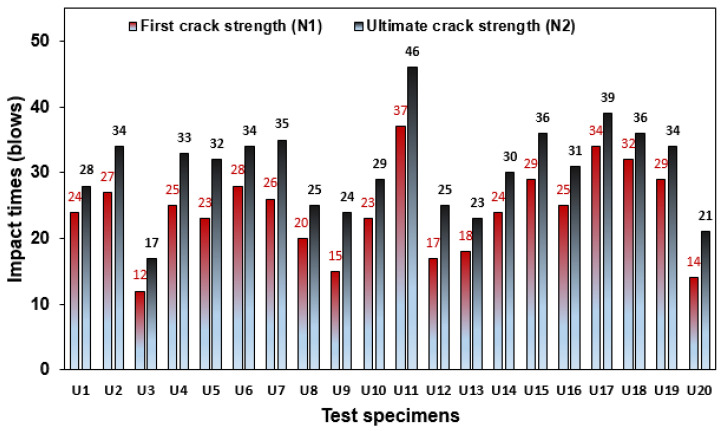
Impact strength of NC-R specimens.

Figure 8 presents the result of 20 reference specimens tested under the DWI test achieved at the N1 and N2 stages. The N1 values among the specimens in the group were in the range of 12 to 36 blows, found in specimens U3 and U11, respectively, as noted in Figure 8. Most of the specimens revealed impact times above 20 blows; the range of impact times was close to each other, indicating less variation in the impact data, which is attributed to the specimen geometry. However, impact data with considerable variation are normally acquired in the multiple drop-weight impact test specified by ACI-2R 544 [37], as reported by many past studies [38,39,40]. Similarly, the impact strength at the N2 stage was in the range of 17 to 46 blows, as shown by specimens U3 and U11.

#### 3.3.2. Impact Strength of NC-to-NC Repaired Specimen

**Figure 9:**
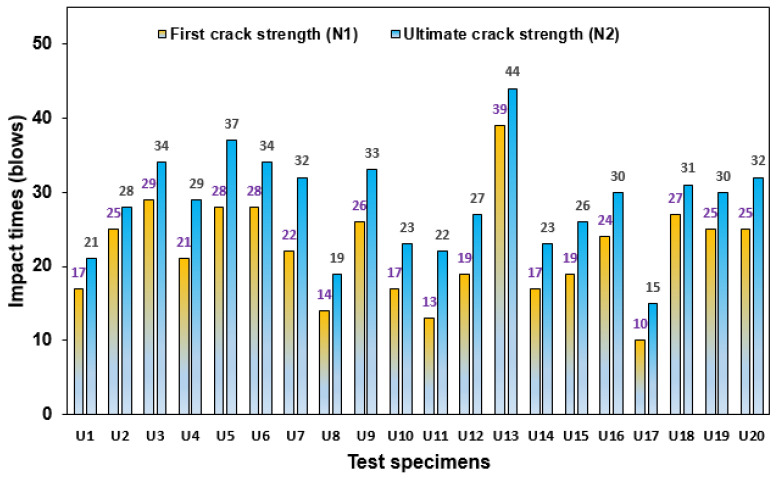
Impact strength of NC-to-NC specimens.

The DWI test results of NC-to-NC repaired specimens under the DWI test are displayed in Figure 9. The individual U-shaped specimen showed an increased strength close to NC-R specimens at the initial crack and complete failure stages. Most of the samples in the group demonstrated an impact load similar to that of the reference specimens. The number of cracking blows for N1 ranged from 10 to 39 blows, exhibited by U17 and U13, respectively. Due to the bonding capability of the PU binder, the U-shaped test specimens also resisted significant impact loads before they underwent complete failure, which is recorded as N2 (see Figure 9). The viscoelastic behavior of polyurethane contributed to the bonding ability to bind the NC substrate together to achieve a monolithic element, which acts as a normal concrete structure. The minimum and maximum N2 values were 15 and 44 blows for specimens U17 and U13, respectively.

### 3.4. Coefficient of Variation (CoV) and Ductility Index (λ) of Test Specimens

**Figure 10:**
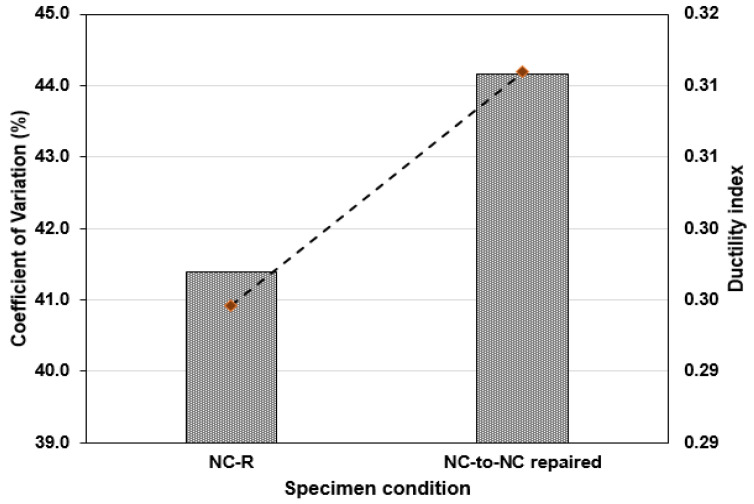
Coefficient of variation and ductility index of test specimens.

The coefficient of variation found among the specimens in the two groups is presented in Figure 10. As shown in Figure 10, NC-R specimens showed lower CoV values than the NC-to-NC repaired specimens. The CoV value of NC-R specimens stands at 41.38%, while NC-to-NC repaired specimens revealed a CoV value of 44.16%, representing an increase in the variation of impact strength data by 19.33%. However, this CoV is less than the values obtained in refs. [41,42].

Additionally, Figure 10 presents the transformation in the ductility index of the U-shaped samples due to specimen conditions. The ductility index (λ) was expressed as the ratio of the number of drops (N1 − N2) to the (N1), enlightening the toughness of the cement-based material after cracking [43,44]. The λ can be obtained using Equation (3).

(3) $$\mathrm{Ductility}\;\mathrm{index}\;(\lambda )=\frac{\left(N2-N1\right)}{N1}$$

In Figure 10, it can be seen that the NC-R specimens displayed a λ-value of 0.295. Due to the bonding effect of PU grout, the ductility index rose to 0.311, describing a change in the concrete’s inherent brittle behavior to a more ductile state. The U-shaped specimens had predefined crack locations, which happened to be at the interface region. The binding agent was characterized by viscoelastic properties, increasing the ductility of the repaired specimens. The reviewed literature [45,46] showed that steel fiber in concrete can also increase its ductility.

### 3.5. Statistical Analysis of Impact Strength Data

#### 3.5.1. Normal and Probability Distribution of NC-R Specimen

**Figure 11:**
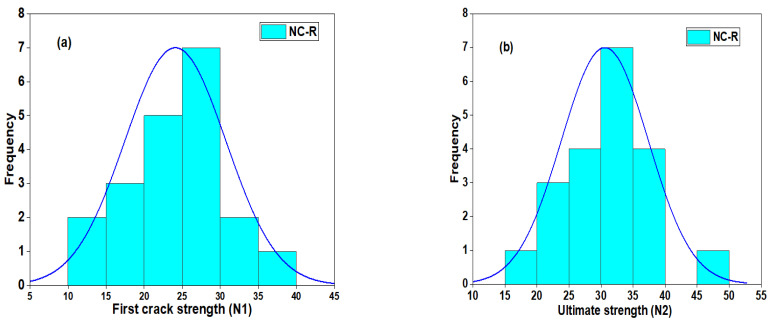
Distribution plot of NC-R specimens (**a**) N1 and (**b**) N2.

**Figure 12:**
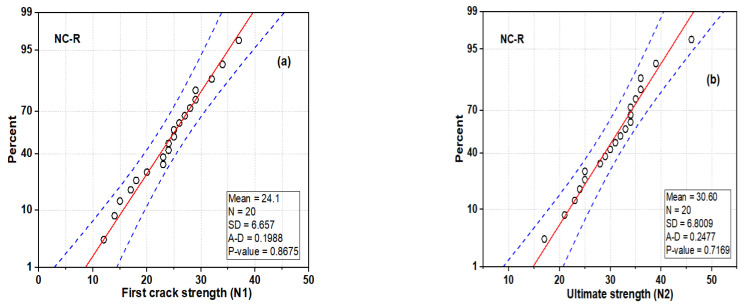
Normal probability plot of NC-R specimens: (**a**) N1 and (**b**) N2.

The distribution plot of the DWI test result at the first crack and the ultimate strength of the NC-R specimens is depicted in Figure 11. Figure 11 shows that the impact strength of N1 and N2 follows a nearly normal distribution, as most of the data points are located with mean values and enclosed with a superimposed distribution curve (see Figure 11a,b). The statistical parameters, including mean, standard deviation, and CoV, are found to be 24 blows, 6.7 blows, and 41.38%, respectively. The ultimate strength (N2) has a mean, standard deviation, and CoV value of 30.6 blows, 6.8 blows, and 44.16%, respectively. The average probability plots of the impact strength data of the NC-R specimen are presented in Figure 12. In Figure 12a,b, it can be observed that data points for N1 and N2 are compacted around the fitting line enclosed within the upper and lower percentile. Moreover, the Anderson Derlin (A-D) test was applied to prove the distribution outcome at a 95% confidence level, and the analysis showed that N1 and N2 had *p*-values = 0.8675 and 0.7169, respectively.

#### 3.5.2. Normal and Probability Distribution of NC-to-NC Repaired Specimens

**Figure 13:**
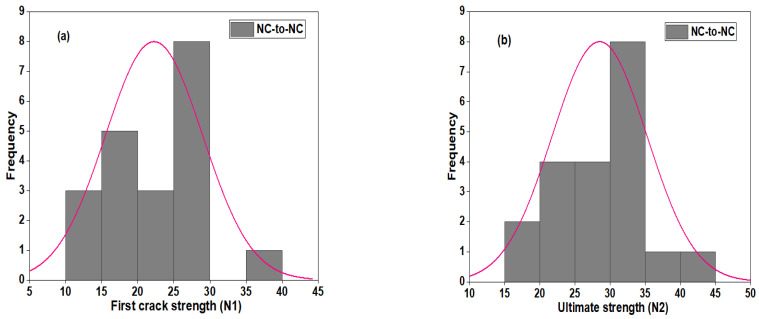
Distribution plot of NC-to-NC repaired specimens: (**a**) N1 and (**b**) N2.

**Figure 14:**
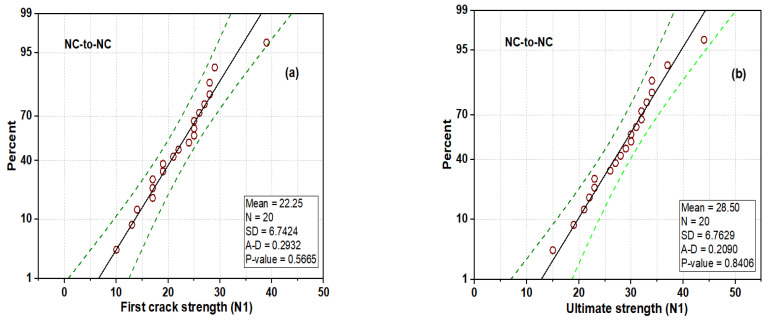
Normal probability plot of NC-to-NC repaired specimens: (**a**) N1 and (**b**) N2.

Figure 13 shows the distribution plot of the DWI test result at the first crack and the ultimate strength of the NC-to-NC repaired specimens. The impact data (N1) barely follow the normal distribution curve, as some data points are situated away from the mean value, as shown in Figure 13a. The impact strength data at the failure stage (N2) nearly follow the distribution, as most of the data points are located with mean value, enclosed with a superimposed distribution curve (see Figure 13b). The statistical parameters, including mean, standard deviation, and CoV, are found to be 22 blows, 6.74 blows, and 41.38%, respectively. The ultimate strength (N2) has a mean, standard deviation, and CoV value of 28.5 blows, 6.763 blows, and 44.16%, respectively. Figure 14 shows the normal probability plots of the impact strength data of NC-to-NC repaired specimens. From Figure 14a,b, it can be observed that data points for N1 and N2 are also compacted around the fitting line enclosed within the upper and lower percentiles. The A-D test was also applied to prove the distribution outcome at a 95% confidence level, and the analysis showed that N1 and N2 had *p*-values = 0.5665 and 0.8406, respectively. The lower *p*-value indicated the scatteredness of the impact strength data at the first crack stage of the repaired U-shaped specimens.

### 3.6. Failure Pattern of the Test Specimen

**Figure 15:**
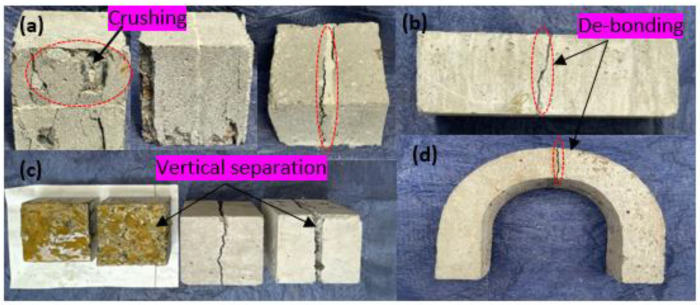
Failure patterns (**a**) crushing of cube, (**b**) de-bonding of beam, (**c**) separation of cube, and (**d**) de-bonding of U-shaped specimens.

Figure 15 depicts the failure modes of the cube, beam, and U-shaped samples under compression, bending, and DWI tests. The cube specimen tested perpendicular to the applied load exhibits the crushing and spalling of concrete at the upper section of the specimen (see Figure 15a). Generally, vertical separation at the interface is the most often observed failure pattern, which happens at the mid-section exhibited by cube-loaded parallel direction with interface plane, beam, and U-shaped specimen, as depicted in Figure 15b–d. However, some specimens revealed a crack that deviated from the interface location. This behavior is also reported by [47]. As shown in Figure 15a, the crack at some cube specimens dissects the bonded PU matrix, while some occur at the surface between the NC substrate and PU matrix.

## 4. Conclusions

In this study, normal concrete specimens, including cubes, beams, and U-shaped specimens, were prepared and cured for 28 days and broken into two parts through natural fracture due to flexural and tensile stresses. The specimens were repaired using a polyurethane matrix by gluing the two parts and tested for compression, splitting, and impact tests to evaluate the bond strength properties. The following conclusion was drawn from the findings.

1. Polyurethane binders effectively repair materials that provide adequate bond strength between the NC substrates, which exceeds the minimum allowable bond strength specified by the ASTM ACI 546-06 for rehabilitating impaired concrete structures.
2. The flexural strength of the reference beam is slightly higher than that of the repaired beam specimen. However, the repaired specimen exhibits a more significant deflection than the NC-R samples. The compressive strength of the NC-to-NC repaired specimens loaded along and parallel to the interface plane revealed a decrease in compressive strength of 47.3% and 31.5% compared to the NC-R samples, respectively.
3. The mean number of blows at the cracking stages appeared nearly equal for reference and repaired NC-to-NC specimens, indicating the effectiveness of the PU matrix in bonding the two pieces together with high strength under repeated impact loads.
4. Common failure patterns were observed for all NC-to-NC repaired specimens, which were characterized by the separation of the two bonded components occurring at interface. However, the cube specimen tested perpendicular to the applied load exhibited the crushing and spalling of concrete at the upper section of the specimen.

## Figures and Tables

**Table 1:** Design mix of the NC mixture (kg/m^3^).

Specimen ID	Cement	Sand	Medium Aggregate	Water
NC	425	718	966	170

**Table 2:** Mixed proportion of PU matrix.

Specimen ID	PU Resin		Diluent (kg/m^3^)
	Polyol (kg/m^3^)	PAPI (kg/m^3^)	
PU matrix	362	60	7.2

**Table 3:** Bond strength of repaired specimen (MPa).

S/N	NC-R	NC-to-NC Specimen	ACI min Acceptable Value
1	6.2	2.5	-
2	6.7	2.9	-
3	6.0	3.0	-
Average	6.3	2.8	1.7–2.1

## Data Availability

The original contributions presented in the study are included in the article, further inquiries can be directed to the corresponding author.
